# Influence of Hot Rolling and Heat Treatment on the Microstructural Evolution of β20C Titanium Alloy

**DOI:** 10.3390/ma10091071

**Published:** 2017-09-12

**Authors:** Xin Liu, Donghui Yu, Qunbo Fan, Ran Shi

**Affiliations:** 1 School of Materials Science and Engineering, Beijing Institute of Technology, Beijing 100081, China; lxlyandccc@163.com (X.L.); eyewind163@163.com (D.Y.); shiranyouleer@163.com (R.S.); 2 National Key Laboratory of Science and Technology on Materials under Shock and Impact, Beijing Institute of Technology, Beijing 100081, China

**Keywords:** β20C titanium alloy, microstructural evolution, EBSD

## Abstract

The microstructural evolution and underlying mechanism of a new high strength, high toughness near β titanium alloy, β20C, during hot deformation, and heat treatment were studied qualitatively and quantitatively. It was found that dynamic recovery occurs mainly in β phase, while α phase undergoes both a dynamic recovery and continuous incomplete dynamic recrystallization with a fraction of high-angle grain boundaries (≥15°) of 21.1% under hot-rolling. Subsequently, α phase undergoes static recrystallization with an increasing fraction of high-angle grain boundaries (21.1%→60.7%) under annealing, while the grains are equiaxed with refined grain sizes of 1.63 µm observed from the rolling direction (RD) and 1.66 µm observed from the transverse direction (TD). Moreover, the average aspect ratio of the lamellar α phase was 2.44 observed from the RD and 3.12 observed from the TD after hot rolling, but decreased to 2.20 observed from the RD, and 2.53 observed from the TD after annealing. Furthermore, the strict Burgers’ relationship between α and β phases changed after hot-rolling and remains the distortion, even after the static recrystallization process of α phase during annealing.

## 1. Introduction

Titanium alloys are attractive materials for many different application fields, owing to their high specific strength and good toughness, combined with corrosion and creep resistance [1,2]. The use of near β titanium alloys is gradually increasing in the aerospace industry (for instance, they are being used in landing gear forgings for airliners) because of their higher yield strength and better fatigue crack growth resistance.

The microstructures and properties of near β titanium alloys are usually sensitive to process parameters. Therefore, the heat treatment and hot deformation related to the optimization of microstructures and properties have received significant attention [3,4,5,6,7,8]. The mechanical properties of Ti-1023 alloy, which was used in landing gear forgings for the Boeing777, have been found to be sensitive to pre-strain and fluctuations in temperature and strain rate [9,10].

Bhattacharjee et al. [11,12] investigated the effect of β grain size on the tensile behavior, ductile fracture toughness, and stress induced martensitic transformation of Ti-1023 alloy. They found that a 0.2% yield stress and the ductile fracture toughness of Ti-1023 alloy exhibited a Hall-Petch relationship with β grain size, and the triggering stress increases with increasing β grain size in a certain range. Fan et al. [13] confirmed that the deformation mechanism of Ti-7333 alloy is dominated by dynamic recovery, as well as α phase globularization in the α + β region, and the globularization trend is more remarkably deformed at higher temperatures and lower strain rates.

Many studies have indicated that the morphological characteristics of a phase largely affect the properties of titanium alloys. Jackson et al. [14] confirmed that the plastic flow behavior of Ti-1023 during isothermal forging is highly dependent on the initial morphology of α phase. Chen et al. [15] studied the microstructural evolution of TC11 during a hot-working process. They noted that the mechanisms of static globularization during the annealing process of TC11 are the static recrystallization of the β-phase, and the static recovery of the α-phase. The processing maps of a new near β titanium alloy, Ti-1300, have been constructed by hot compression testing and the results revealed a rheological phenomenon that leads to Ti-1300 instability, easily occurring at high strain rates [16].

Jones et al. [17] investigated the flow behavior and microstructural evolution of Ti-5553 during subtransus isothermal forging. The results show that the initial flow behavior and dominating deformation mechanism depended upon the morphology and volume fraction of the α precipitates. Zhao et al. [18] found that the flow softening behavior is related to the globularization of the acicular microstructure and deformation heating during isothermal forging of the Ti-17 powder compact. Warchomicka et al. [19] studied the hot deformation behavior near to the β transus temperature of Ti-5Al-5Mo-5V-3Cr-1Zr alloy by using the electron backscatter diffraction (EBSD) technique. They illustrated that the deformation parameters have a significant effect on dynamic recrystallization, orientation, and the type of grain boundary. Numerous studies have shown that heat treatment and hot deformation have a remarkable influence on the microstructural evolution and mechanical properties of titanium alloys. In addition, the application of additive manufacturing techniques for fabrication of titanium parts has been widely considered by numerous researchers. Razavi et al. [20,21] made positive efforts in the selective laser melting of Ti-6Al-4V.

β20C is a new high strength, high toughness near β titanium alloy developed at the Beijing Institute of Technology. The β transus for this material is about 900 °C. Its chemical composition is given in Table 1. It exhibits an exceptional strength and elongation characteristics. This material can exhibit a tensile strength of 1200 MPa (300 MPa more than Ti6-Al4-V) and an elongation of 15% (33% more than Ti6-Al4-V). The microstructure consists of fine equiaxed grains of 1–3 µm obtained after hot deformation and heat treatment, while the grain sizes of conventional titanium alloys are usually 10 µm or so via a similar process. There are close relationships between the properties of β20C and its microstructure. Therefore, further research is required to understand its mechanisms of microstructural evolution and the orientation relationship between the α and β phases during hot deformation and heat treatment. Furthermore, a quantitative analysis of the orientation relationship is also required.

## 2. Experimental

The experiments were conducted using samples of β20C alloy. An as-cast β20C ingot was heated at 900 °C for 1 h and then rolled into a plate with a reduction of 78.6% (70 mm→15 mm) by eight passes within a temperature range of 700–800 °C, followed by air-cooling. Subsequently, the rolled β20C plate was annealed at 850 °C for 2 h followed by furnace cooling. Lamellar specimens were machined with dimensions of 6 × 4 × 2 mm^3^ from the as-cast, hot-rolled, and annealed materials.

Observations were made on a part of the specimens etched with a solution of 2% HF, 10% HNO_3_, and 88% H_2_O after mechanical polishing. Other parts of the specimens were electropolished (25 °C, 25 V, ~30 s, solution: 6% HClO_4_, 34% CH_3_(CH_2_)_3_OH, and 60% CH_3_OH) to eliminate the surface stress in order to achieve the surface quality required for EBSD examinations. EBSD was carried out to characterize the as-cast, hot-rolled, and annealed microstructures of the alloys by using FEI Nova NanoSEM 430 field emission gun scanning electron microscope operated at 20 kV produced by FEI, Hillsboro, MO, USA.

**Figure 1:**
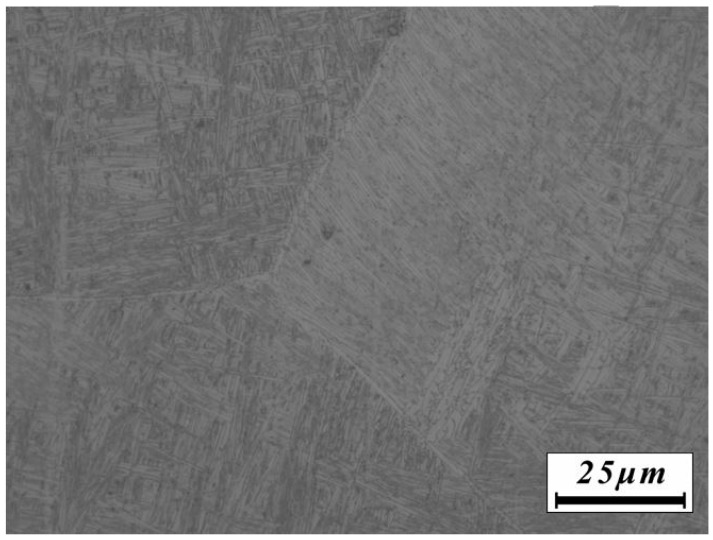
Original microstructure of the as-cast β20C titanium alloy.

The β20C alloy in this study was produced by thrice melting in a vacuum consumable melting furnace. Its original as-cast microstructure is shown in Figure 1. The Widmanstätten structure is comprised of large β grains with an average grain size of 1–2 mm and a large number of fine α lamellae. The α lamellae are distributed continuously at grain boundaries separating β grains with specific misorientations. Some fine α phase nucleate at grain boundaries and grow into the inner areas of the β grains.

**Figure 2:**
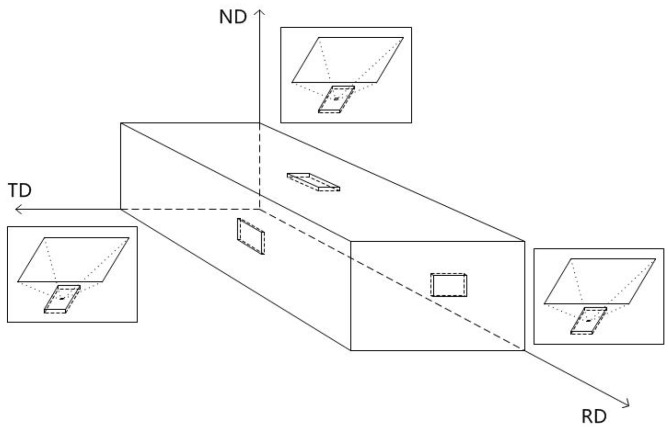
Schematic showing the sampling after hot rolling and annealing.

As Figure 2 shows, all of the hot-rolled and annealed specimens were cut along the normal direction (ND), the rolling direction (RD), and the transverse direction (TD), respectively. The specimen was observed from the corresponding direction.

Tensile specimens were cut along the RD and the TD. According to GB/T 228-2002 (2002) Metallic materials-Tensile testing at ambient temperature (2002) [22], tensile tests were performed on Instron 5985 testing machine produced by Instron^®^, Norwood, MA, US at room temperature, at a strain rate of 10^−3^/s.

## 3. Results and Discussion

### 3.1. Microstructural Evolution

**Figure 3:**
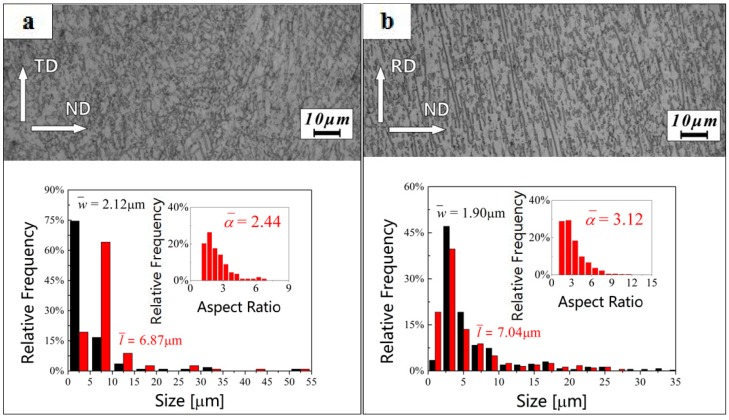
Statistics of the grain size distribution in the lamellar α phase and the microstructure of hot-rolled β20C observed from the (**a**) rolling direction (RD) and (**b**) transverse direction (TD).

The microstructures of the hot-rolled β20C alloy and the statistical relative frequency distribution of the grain size and aspect ratio in α phase are shown in Figure 3. Using quantitative metallographic analysis software Image Pro Plus v6.0 produced by Media Cybernetics, Washington, D.C., USA, several hundreds of grains were counted. It is found that the average aspect ratio $\left(\bar{\alpha }\right)$ of the lamellar α grains is 2.44 observed from the RD (Figure 3a) and 3.12 observed from the TD (Figure 3b), and the directions of the α grains are obviously parallel to the RD. After hot rolling, α phase becomes sparser and coarser. Moreover, the α grains are elongated along the RD, with the mean width ($\bar{w}$) and length ($\bar{l}$) of 2.12 µm and 6.78 µm observed from the RD or 1.90 µm and 7.04 µm observed from the TD.

**Figure 4:**
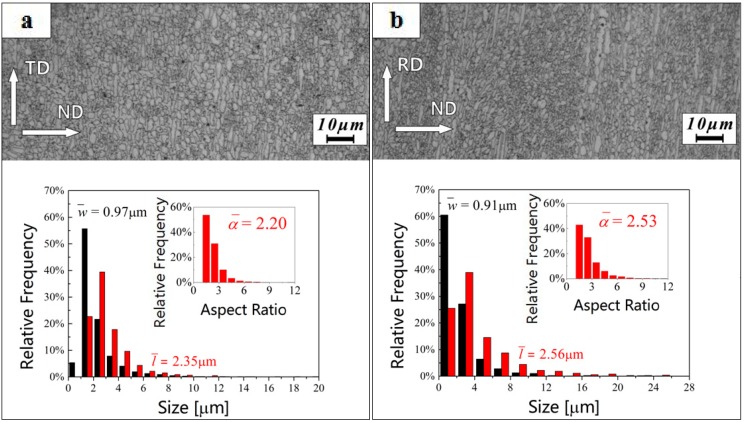
Statistics of the grain size distribution in the lamellar α phase and the microstructure of annealed β20C observed from the (**a**) RD and (**b**) TD.

The microstructures of the annealed β20C alloy and the statistical distribution of the grain size, and the aspect ratio in the α phase are shown in Figure 4. It is seen that the α phase undergoes static recrystallization during annealing and becomes more globular with an average aspect ratio $\left(\bar{\alpha }\right)$ of 2.20 observed from the RD (Figure 4a) and 2.53 observed from the TD (Figure 4b). The morphology of some α grains retains roughly lamellar structures and the directions of the α grains are partly parallel to the RD. Moreover, the average α grain size is 1.63 µm observed from the RD or 1.66 µm observed from the TD. The results indicate that the α grains are equiaxed and refined during annealing.

### 3.2. EBSD Microstructure

**Figure 5:**
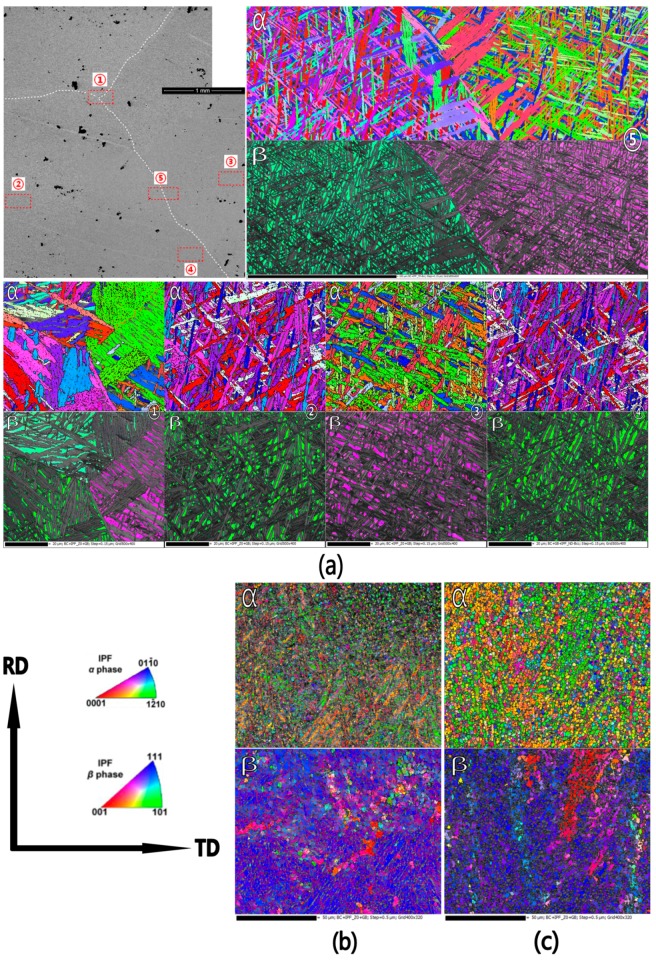
Orientation maps of the (**a**) as-cast, (**b**) hot-rolled, and (**c**) annealed β20C alloys.

**Figure 6:**
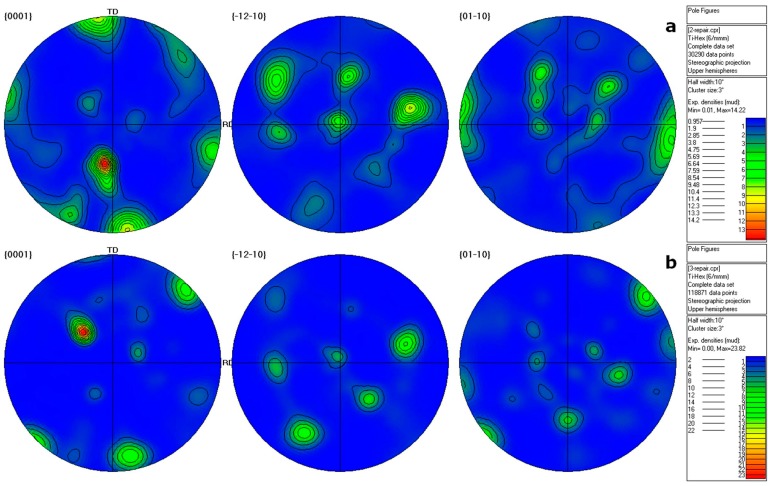
Pole figures of the (**a**) hot-rolled and (**b**) annealed β20C alloys.

Figure 5 shows the microstructural orientation maps of the α phase and β phase in the as-cast (Figure 5a), hot-rolled (Figure 5b), and annealed (Figure 5c) β20C as observed from the ND. The corresponding pole figures are shown in Figure 6. Figure 5a displays the orientation maps of five typical regions (①~⑤) of large grains in the as-cast β20C. It can be seen that the α phase shows a crisscross type pattern within the β grain. Statistics indicate that the fractions of α phase and β phase are 82.5% and 17.5%, respectively. Meanwhile, this is consistent with the characteristic of the as-cast β20C (Figure 1). After hot rolling, the fractions of α phase and β phase are found to be 23.7% and 76.3%, respectively. It can be inferred that most α phase dissolved into β phase with the heating before rolling. A large number of original grains fragment into misoriented small domains during hot rolling, and substructures form inside (Figure 5b). Grains elongate along the RD with serrated boundaries and show a typical dynamic recovery feature. Moreover, some fine dynamic recrystallization grains nucleate at boundaries. This indicates that incomplete dynamic recrystallization occurred for hot rolling while the texture forms. There is a relatively high concentration of {01−10} poles in the RD (Figure 6a). During annealing, the elongated grains nearly disappear and the growth of recrystallization grains occurs (Figure 5c). This illustrates that static recrystallization occurs mainly in this situation. With the recrystallized α grains growing, the fractions of α phase increases to 59%, and the fractions of β phase decreases to 41%. In addition, the texture strengthens during annealing (Figure 6c).

### 3.3. Misorientation Statistical Analysis

**Figure 7:**
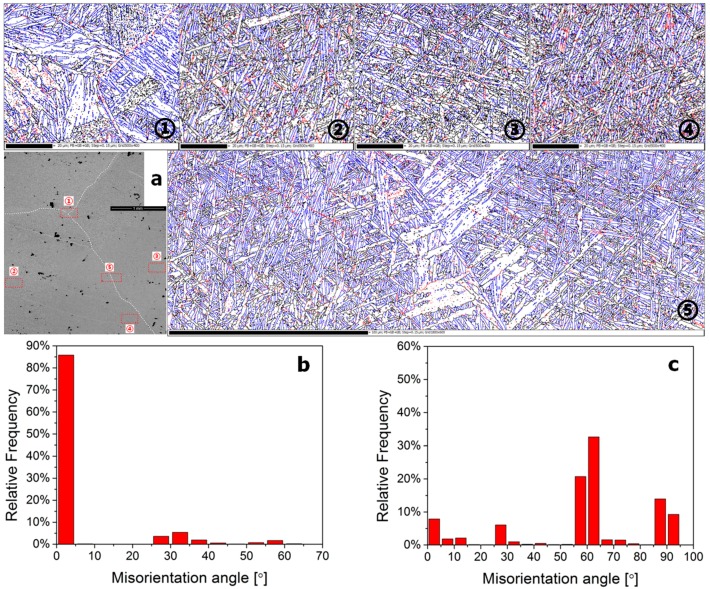
Electron backscatter diffraction (EBSD) grain boundary map (**a**) and corresponding misorientation distributions of the as-cast β20C alloy in β (**b**) and α (**c**) phases.

**Figure 8:**
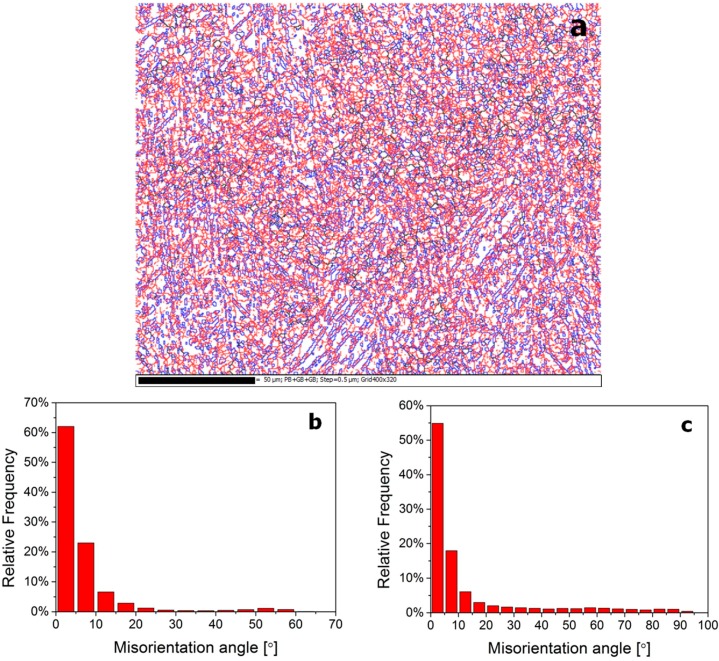
EBSD grain boundary map (**a**) and corresponding misorientation distributions of the hot-rolled β20C alloy in β (**b**) and α (**c**) phases.

**Figure 9:**
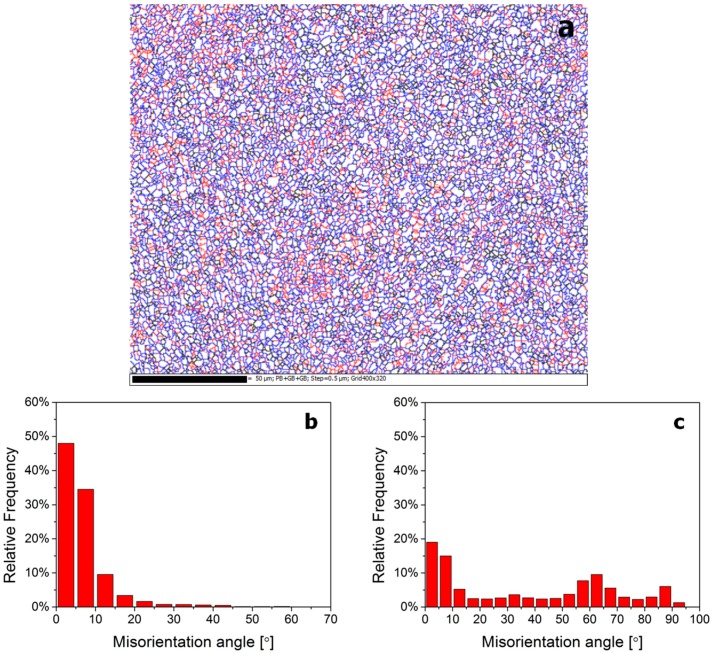
EBSD grain boundary map (**a**) and corresponding misorientation distributions of the annealed β20C alloy in β (**b**) and α (**c**) phases.

Figure 7, Figure 8 and Figure 9 show the misorientation angle distributions of all possible grain boundaries, and the corresponding EBSD grain boundary map. Low-angle grain boundaries (<15°, LAGBs), high-angle grain boundaries (≥15°, HAGBs), and phase boundaries are depicted as red, black, and blue lines in Figure 7a, Figure 8a and Figure 9a, respectively.

Based on the statistics derived from Figure 7a, the misorientation angle distributions of grain boundaries in the β20C as-cast microstructure, displayed by the histogram in Figure 7b,c. It can be seen that the fraction of LAGBs is 87.0% in β phase (Figure 7b) and the fraction of HAGBs is 88.2% in α phase (Figure 7c). In addition, the transitions from LAGBs to HAGBs are discontinuous in both the β-phase and α-phase misorientation distributions. The grain boundary maps of five typical regions (①~⑤) of large grains in the as-cast β20C are displayed, and the results are consistent with known features of the β20C as-cast microstructure (Widmanstätten structure).

Based on the statistics derived from Figure 8a, the misorientation angle distributions of grain boundaries in the β20C hot-rolled microstructure can be obtained, which are displayed by the histogram in Figure 8b,c. It can be seen that the fraction of LAGBs is 91.6% in β phase (Figure 8b) and the transition from LAGBs to HAGBs is relatively continuous as compared with Figure 7b. It is suggested that the dynamic recovery mainly occurs and the original grains fragment into misoriented small domains during hot rolling. Moreover, the fraction of HAGBs in α phase is also lower (21.1%) and the transition from LAGBs to HAGBs becomes continuous obviously (Figure 8c), when compared with Figure 7c. However, the phase boundaries show some “new grains” with small size distributed along the grain boundaries, and a part of them formed a necklace structure, which is usually identified as one of the characteristics of recrystallization (Figure 8a). It is indicated that α phase undergoes both a dynamic recovery and a continuous dynamic recrystallization during hot rolling.

Based on the statistics derived from Figure 9a, the misorientation angle distributions of grain boundaries in annealed β20C microstructure can be obtained, which are displayed in the histogram in Figure 9b,c. It can be seen that the misorientation distribution of β phase (Figure 9b, 92.1% LAGBs) is almost the same as that in hot rolled β20C (Figure 8b, 91.6% LAGBs), except for the more uniform distribution of LAGBs. This illustrates that only static recovery takes place in β phase during annealing after hot rolling. In comparison with the misorientation distribution of α phase in hot-rolled β20C (Figure 8c), the fraction of HAGBs in α phase increases dramatically to 60.7% and the fraction of LAGBs becomes smaller (Figure 9c) with the approximate pattern of the misorientation distribution map. Moreover, the phase boundaries increase in its fraction as compared with that in the hot-rolled microstructure. They also show significant growth and equiaxed of the recrystallized α grains. It is suggested that static recrystallization and volume growth occurs in α phase during annealing after hot rolling.

### 3.4. Orientation Relationship between α and β Phases

**Figure 10:**
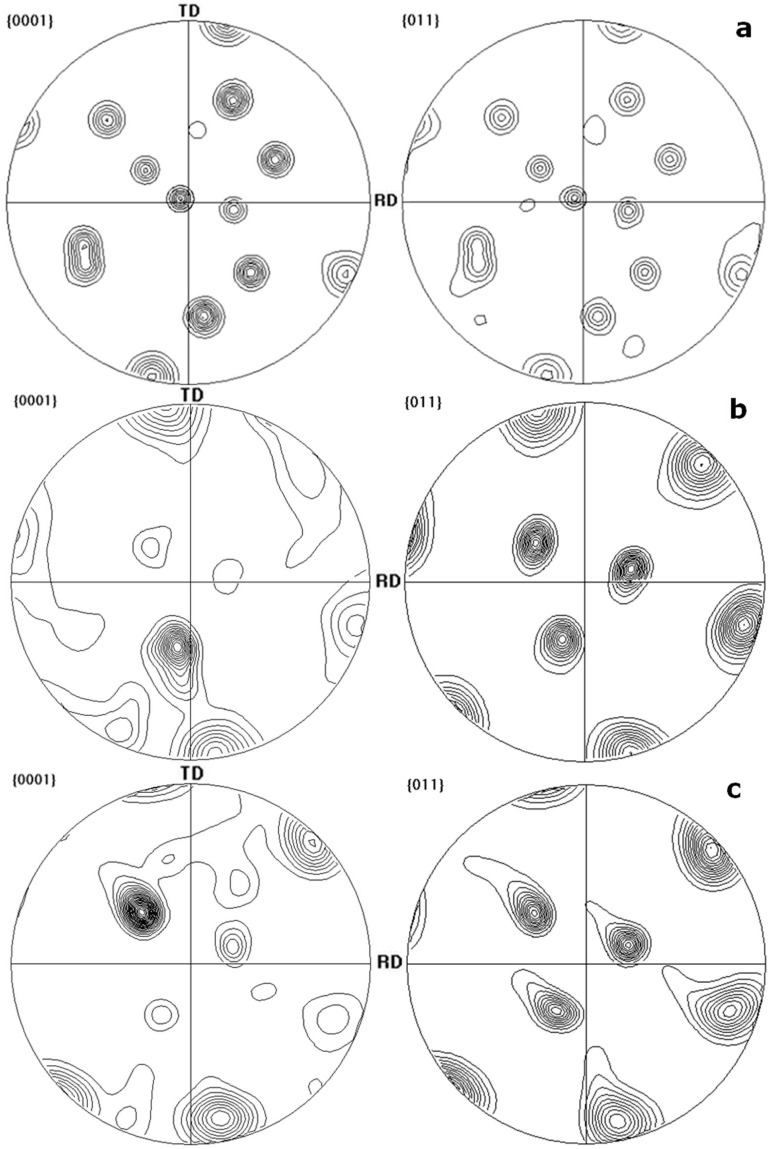
Pole figures of the (**a**) as-cast, (**b**) hot-rolled and (**c**) annealed β20C alloys.

The crystallography of α and β phases in titanium alloys under no deformation conditions has been investigated in depth [23,24]. Generally, the orientation variants resulting from the β-α phase transformation, according to Burgers relationship, are displayed in Table 2 [13]. This is consistent with the orientation relationship of α/β in the as-cast β20C alloys, as indicated by the pole figures in Figure 10a.

Figure 10b shows the pole figures of the corresponding crystal plane groups after hot rolling. They illustrate that there is no strict Burgers relationship between α and β phases, meaning the orientation relationship of α/β has changed after hot-rolling. It may be ascribed to the inhomogeneity in the strain distribution and the increased α/β interface energy in hot working. Figure 10c shows the pole figures of the corresponding crystal plane groups after annealing. They illustrate that there is also no strict relationship between α and β phases, meaning that the orientation relationship of α/β remains its distortion in a certain degree even after the static recrystallization process of the α phase.

### 3.5. Mechanical Properties

**Figure 11:**
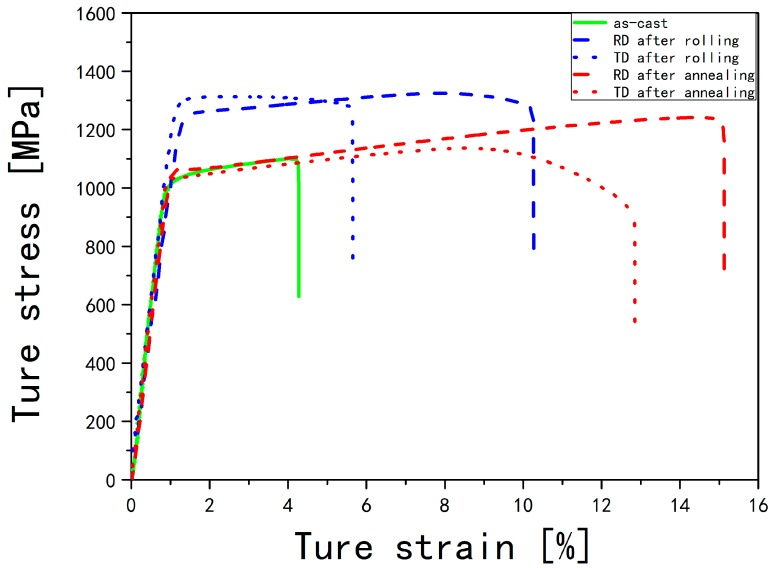
True stress-strain curves for β20C alloys at a strain rate of 10^−3^/s in the RD and the TD.

The true stress-strain curves of the as-cast, hot-rolled, and annealed β20C alloys at room temperature, at a strain rate of 10^−3^/s in the RD and the TD are shown in Figure 11.

It can be seen that the as-cast β20C alloy has a poor strength and ductility. The ultimate tensile strength (1096 MPa) and elongation (4.13%) of the as-cast β20C are both the smallest. After hot-rolling, both the strength and ductility increased obviously. Especially in the RD, the hot-rolled β20C has a good elongation (10%) with a greatest strength (1332 MPa). Noticeably, the annealed β20C has a good balance of strength and ductility in the RD with a moderate ultimate tensile strength (1202 MPa) and the greatest elongation (14.8%). In addition, the ductility in the RD is always better than that in the TD under the same conditions.

## 4. Conclusions

The formation of fine equiaxed grains in β20C alloy obtained after hot deformation and heat treatment has been investigated by optical metallographic observation and EBSD. The following principal conclusions can be drawn from the present study:

(1) Indicated by the microstructural orientation maps and histograms of misorientation distributions obtained from EBSD data, dynamic recovery occurs mainly in the β phase, and α phase undergoes both dynamic recovery and continuous incomplete dynamic recrystallization with the fraction of HAGBs at 21.1% under hot-rolling while the texture forms. The original β grains fragment into misoriented small domains, and the α grains are elongated along the RD, becoming sparser and coarser. In the optical microstructural maps, the average aspect ratio $\left(\bar{\alpha }\right)$ of the lamellar α phase is found to be 2.44 observed from the RD and 3.12 observed from the TD.
(2) Under annealing, the static recovery takes place mainly in β phase, and α phase undergoes static recrystallization with an increasing fraction of HAGBs (21.1%→60.7%). The α grains are equiaxed with the refined grain sizes of 1.63 µm, as observed from the RD and 1.66 µm observed from the TD. In addition, it is shown by the pole figures that the texture strengthens with a relatively high concentration of {0001} poles.
(3) Comparing the pole figures of the corresponding crystal plane groups after hot rolling, the orientation relationship of α/β in β20C are found to be changed after hot rolling. There was no longer a strict Burgers relationship between α and β phases even after the static recrystallization process of α phase during annealing.
(4) The as-cast β20C alloy has the smallest ultimate tensile strength (1096 MPa) and elongation (4.13%). In the RD, β20C has a greatest strength (1332 MPa) after hot-rolling and a greatest elongation (14.8%) after annealing. The ductility in the RD is always better than that in the TD under the same conditions.

## Figures and Tables

**Table 1:** Chemical composition (wt %) of the β20C alloy.

Alloy Element	Al	Cr	Mo	Fe	Zr	Sn	Zn	O	N	H	C
wt %	5.12	2.5	4.48	0.52	1.8	1.1	2.9	0.08	0.02	0.002	0.01

**Table 2:** Orientation variants resulting from the β→α phase transformation according to Burgers relationship.

Variant Number	Corresponding Plane (hkl)β||(hkil)α	Corresponding Direction [uvw]β||[uvtw]α
1	(011)β||(0001)α	[−1−11]β||[2−1−10]α
2	-	[1−11]β||[2−1−10]α
3	(−101)β||(0001)α	[1−11]β||[2−1−10]α
4	-	[111]β||[2−1−10]α
5	(0−11)β||(0001)α	[111]β||[2−1−10]α
6	-	[−111]β||[2−1−10]α
7	(101)β||(0001)α	[−111]β||[2−1−10]α
8	-	[−1−11]β||[2−1−10]α
9	(−110)β||(0001)α	[−1−11]β||[2−1−10]α
10	-	[111]β||[2−1−10]α
11	(110)β||(0001)α	[1−11]β||[2−1−10]α
12	-	[−111]β||[2−1−10]α
